# Morpho-Functional 1H-MRI of the Lung in COPD: Short-Term Test-Retest Reliability

**DOI:** 10.1371/journal.pone.0137282

**Published:** 2015-09-01

**Authors:** Bertram J. Jobst, Mark O. Wielpütz, Simon M. F. Triphan, Angela Anjorin, Julia Ley-Zaporozhan, Hans-Ulrich Kauczor, Jürgen Biederer, Sebastian Ley, Oliver Sedlaczek

**Affiliations:** 1 Department of Diagnostic & Interventional Radiology, University Hospital of Heidelberg, Heidelberg, Germany; 2 Translational Lung Research Center Heidelberg (TLRC), Member of the German Lung Research Center (DZL), Heidelberg, Germany; 3 Department of Diagnostic and Interventional Radiology with Nuclear Medicine, Thoraxklinik at University of Heidelberg, Heidelberg, Germany; 4 Research Center Magnetic Resonance Bavaria (MRB), Würzburg, Germany; 5 Institute for Clinical Radiology, Ludwig-Maximilians-University Hospital Munich, Munich, Germany; 6 Radiologie Darmstadt, Department of Radiology, County Hospital Gross-Gerau, Gross-Gerau, Germany; 7 Department of Diagnostic & Interventional Radiology, Surgical Hospital Dr. Rinecker, Munich, Germany; Technion—Israel Institute of Technology, ISRAEL

## Abstract

**Purpose:**

Non-invasive end-points for interventional trials and tailored treatment regimes in chronic obstructive pulmonary disease (COPD) for monitoring regionally different manifestations of lung disease instead of global assessment of lung function with spirometry would be valuable. Proton nuclear magnetic resonance imaging (1H-MRI) allows for a radiation-free assessment of regional structure and function. The aim of this study was to evaluate the short-term reproducibility of a comprehensive morpho-functional lung MRI protocol in COPD.

**Materials and Methods:**

20 prospectively enrolled COPD patients (GOLD I-IV) underwent 1H-MRI of the lung at 1.5T on two consecutive days, including sequences for morphology, 4D contrast-enhanced perfusion, and respiratory mechanics. Image quality and COPD-related morphological and functional changes were evaluated in consensus by three chest radiologists using a dedicated MRI-based visual scoring system. Test-retest reliability was calculated per each individual lung lobe for the extent of large airway (bronchiectasis, wall thickening, mucus plugging) and small airway abnormalities (tree in bud, peripheral bronchiectasis, mucus plugging), consolidations, nodules, parenchymal defects and perfusion defects. The presence of tracheal narrowing, dystelectasis, pleural effusion, pulmonary trunk ectasia, right ventricular enlargement and, finally, motion patterns of diaphragma and chest wall were addressed.

**Results:**

Median global scores [10(Q1:8.00;Q3:16.00) vs.11(Q1:6.00;Q3:15.00)] as well as category subscores were similar between both timepoints, and kappa statistics indicated “almost perfect” global agreement (ĸ = 0.86, 95%CI = 0.81–0.91). Most subscores showed at least “substantial” agreement of MRI1 and MRI2 (ĸ = 0.64–1.00), whereas the agreement for the diagnosis of dystelectasis/effusion (ĸ = 0.42, 95%CI = 0.00–0.93) was “moderate” and of tracheal abnormalities (ĸ = 0.21, 95%CI = 0.00–0.75) “fair”. Most MRI acquisitions showed at least diagnostic quality at MRI1 (276 of 278) and MRI2 (259 of 264).

**Conclusion:**

Morpho-functional 1H-MRI can be obtained with reproducible image quality and high short-term test-retest reliability for COPD-related morphological and functional changes of the lung. This underlines its potential value for the monitoring of regional lung characteristics in COPD trials.

## Introduction

At present, pulmonary function testing is the method of choice for initial diagnosis and follow-up of chronic obstructive pulmonary disease [1]. As manifestations of the disease are typically inhomogeneous with different degrees of involvement throughout the lung and as compensation of functional impairment of the diseased areas by intact portions of the organ is relevant, global parameters from PFT such as FEV1% may not be optimally suitable for monitoring disease activity and the efficacy of treatment [1]. Due to the evolving spectrum of therapeutic options for COPD patients, requirements related to the monitoring of lung structure and function in interventional COPD trials are increased. Alternative endpoints which reflect changes in regional lung structure and function would be most welcome. Many requirements in this field are met by computed tomography (CT), which is the current modality of choice for in vivo imaging of COPD [2], but longitudinal follow-up with CT cannot be achieved without excess radiation exposure [3]. In recent years proton MRI has emerged as a promising new option for the radiation-free characterization and follow-up of COPD [4–6]. Current MRI techniques comprise morphological as well as functional sequences combining reasonable diagnostic accuracy with relatively easy handling [7]. In this context, MRI-based monitoring of patients with cystic fibrosis demonstrated the ability of MRI to depict alterations of lung structure and perfusion, and detect the response to therapy in patients with exacerbation [8]. MRI perfusion imaging has already been shown to be able to distinguish patients with mild COPD from healthy controls [9], and to correlate well with both FEV1% [10] and diffusing capacity [11]. But the applicability of imaging procedures in the context of patient monitoring not only depends on diagnostic accuracy and easy handling, but also on reproducibility. Test-retest reliability of imaging-derived biomarkers such as scores for morphologic changes and perfusion needs to be assessed before their use as trial endpoints in the scientific or clinical context. The aim of this study was to shape a streamlined MRI protocol based on commercially (“out of the box”) available sequences which provides a comprehensive assessment of both morphological and functional lung characteristics of COPD patients within a single study and evaluate its short-term test-retest reliability. For this purpose, 20 COPD patients of different GOLD stages underwent MRI on two consecutive days, and all exams were assessed using an established MRI score for airways disease.

## Methods

### Ethics statement

This prospective study was approved by the Institutional Review Board of the Medical Faculty of the University of Heidelberg, Germany. The work was carried out in accordance with the Declaration of Helsinki (The Code of Ethics of the World Medical Association). Prior to participation in the study, all subjects gave their written informed consent.

### Study design and patient characteristics

N = 20 patients [median age 66 (Q1:59; Q3:74) years] diagnosed with COPD and different disease severity according to the GOLD criteria [12] were enrolled. The cohort included n = 10 patients with mild COPD (GOLD stages I-II) recruited from an outpatient clinic, and n = 10 patients with severe COPD (GOLD stages III-IV) from a specialized chest clinic (Table 1). Each patient prospectively underwent an identical lung MRI exam on two consecutive days (MRI1 / MRI2).

**Table 1:** Patient characteristics. 10 patients with mild COPD (GOLD stages I & II) and 10 patients with severe COPD (GOLD stages III & IV) were enrolled into the study.

	GOLD I & II	GOLD III & IV	Global
N	10	10	20
Male / Female (n)	9/1	9/1	18/2
Min age (y)	49	60	49
Max age (y)	77	79	79
Median age (y)	59 (Q1:55; Q3:70)	69 (Q1:63; Q3:74)	66 (Q1:59; Q3:74)

### Imaging

The MRI protocol was specifically tailored to the demands of structural and functional alterations of the pulmonary parenchyma, vasculature and airways using a clinical 1.5T scanner (Magnetom Avanto, Siemens Medical Solutions, Erlangen, Germany) and current protocol recommendations based on commercially available sequences [7]. Structural aspects were addressed with the following sequences in coronal and transverse orientation: Volume interpolated gradient echo (GRE, VIBE), acquired in inspiratory breath-holds, before and after contrast application (incl. fat saturation), fat-saturated, free-breathing periodically rotated overlapping parallel lines with enhanced reconstruction (BLADE), acquired in free breathing and triggered to the expiratory phase, half-fourier acquisition single-shot turbo spin-echo (HASTE) in inspiratory breath-holds, and balanced steady-state free precession (bSSFP, TrueFISP) during free breathing. Breath-holding times of most morphological sequences were kept below 25 seconds, except VIBEs that include fat saturation pulses. Dynamic contrast enhanced (DCE) first pass perfusion MRI was based on a three-dimensional GRE view sharing sequence with stochastic trajectories (TWIST) at high temporal resolution (1.47 s/volume) during inspiratory breath-hold and contrast bolus injection by a power injector (0.05 mmol/kg Gd-DTPA, Magnevist, Bayer Schering Pharma AG, Berlin, German) at a rate of 5 ml/s followed by a bolus chaser of 30 ml NaCl). The acquisition comprised 24 consecutive measurements, covering a time span of max. 37s. The initial passage of the contrast bolus through the pulmonary vasculature required a breath-holding time of 15–25 seconds. Patients were advised to hold breath as long as possible and then breathe shallowly. Subtraction images in coronal orientation with a spatial resolution of 1.95 x 1.95 mm in-plane and 5 mm slice thickness were used for visual analysis. For the evaluation of respiratory mechanics, two-dimensional time-resolved TrueFISP (dynamic TrueFISP) sequences were acquired during maximum inspiration and expiration in coronal and angulated sagittal orientation, each through the center of the right and left diaphragm. In total, image acquisition took on average 30 minutes. Details on the applied sequence parameters are provided in Table 2.

**Table 2:** MRI protocol and sequence parameters.

sequence	mode	orien-tation	breathmode	contrast medium	TR (ms)	TE (ms)	FoV (mm^2^)	slice thickn. (mm)	voxel size (mm^2^)	matrix	scan time (min:s)
TrueFISP	2D	cor	free	native	339.29	1.16	500×500	4.0	19.5×1.95	256×256	0:43
TrueFISP	2D	tra	free	native	290.30	1.16	440×358	4.0	1.95×1.95	256×208	0:37
VIBE	3D	cor	insp	native	3.20	1.14	500×500	4.0	0.98×0.98	256×256	0:20
VIBE	3D	tra	insp	native	3.46	1.18	400×350	4.0	1.56×1.56	256×224	0:23
HASTE	2D	cor	insp	native	600	32.00	500×500	6.0	0.98×0.98	512×512	0:13×2
HASTE	2D	tra	insp	native	571	42.00	400×350	6.0	1.56×1.56	256×224	0:18
BLADE	2D	cor	trig	native	6300.25	83.00	500×500	4.0	1.56×1.56	320×320	2:49
BLADE	2D	tra	trig	native	7048.25	83.00	500×500	4.0	1.56×1.56	320×320	3:42
TWIST	4D	cor	insp	dynamic	1.80	0.68	500×500	5.0	1.95×1.95	256×256	0:37
VIBE FatSat	3D	cor	insp	post-contrast	3.35	1.18	500×500	4.0	0.98×0.98	512×512	0:33
VIBE FatSat	3D	tra	insp	post-contrast	3.35	1.18	400×350	4.0	0.78×0.78	512×448	0:32
TrueFISP	2D+t	cor	forced	post-contrast	317.10	1.08	500×500	6.0	0.98×0.98	512×512	0:24
TrueFISP	2D+t	sag R	forced	post-contrast	317.10	1.11	450×450	6.0	0.88×0.88	512×512	0:25
TrueFISP	2D+t	sag L	forced	post-contrast	317.10	1.11	450×450	6.0	0.88×0.88	512×512	0:25

All sequences are acquired in 3D or 2D multi-slice modes and were accelerated using GRAPPA with a parallelization factor of 2. Breathing mode indicates **free** breathing, one or more breath-hold in **insp**iration or **exp**iration, expiration **trig**gered acquisition or dynamic acquisition during **forced in**- and **exp**iration.

### Image evaluation

MRI1 and MRI2 were evaluated employing a scoring system specifically reporting the extent and distribution of COPD-related abnormalities, which was a modification of a previously established MRI scoring system for airways disease [8,13]. The scoring system categorizes COPD-related abnormalities, and each category is rated with a 3-point scale of 0, 1 or 2 or a 2-point scale of 0 and 1 (max. score 98) in a lobe-based approach as described in the following:

#### Tracheal disease

For the diagnosis of tracheal disease, we considered conditions affecting the structure of the trachea or right and left stem bronchi such as collapse, stenosis, or saber sheath trachea. The rating of tracheal pathologies was mainly based on inspiratory VIBE, BLADE, and TrueFISP. The BLADE images were acquired during expiratory phase and thus provided complementary information to inspiratory VIBE images. Together, the previously mentioned sequences allowed for the visual evaluation of the tracheal cross-sectional area during the breathing cycle: 0 = absent, 1 = moderate (≤ 50% decrease in lumen area), 2 = severe (> 50% decrease in lumen area).

#### Bronchial disease

This category summarizes COPD-related pathologies with localisation in the lobar bronchi and segmental bronchi in a combined approach [13], i.e. bronchiectasis, bronchial wall thickening, stenosis, and bronchial mucus plugging with hyperintense intraluminal appearance on T2-weighted images. 0 = absent, 1 = ≤50% of lobe, 2 = >50% of lobe.

#### Small airway disease

Tree-in-bud appearance and peripheral bronchiectasis (visibility of bronchi within 2 cm from the pleural surface): 0 = absent, 1 = ≤50% of lobe, 2 = >50% of lobe.

#### Consolidations

Areas with an increase in parenchymal signal intensity > 2.0 cm in diameter with possible presence of air bronchogram or contrast enhancement [13]: 0 = absent, 1 = ≤50% of lobe, 2 = >50% of lobe.

#### Nodules

0 = absent, 1 = < 1.0 cm, 2 = ≥ 1.0 cm.

#### Parenchymal defects

Areas of decrease in parenchymal signal intensity on inspiratory HASTE, VIBE, and/or post-contrast VIBE [14]: 0 = absent, 1 = ≤50% of lobe, 2 = >50% of lobe.

#### Perfusion defects

Areas of reduced or missing enhancement on subtracted peak perfusion maps. Other causes not related to airway disease or emphysema, in particular pulmonary embolism, infiltration and atelectasis, were excluded [13]: 0 = absent, 1 = ≤50% of lobe, 2 = >50% of lobe.

#### Perfusion defects due to collateral causes

Perfusion defects not related to airway disease (infiltration, atelectasis, pulmonary embolism): 0 = absent, 1 = ≤50% of lobe, 2 = >50% of lobe.

#### Impaired respiratory mechanics

Motion impairment of the chest wall and/or diaphragm such as diaphragmatic depression with / without paradoxical motion as published previously [15–17]. Different causes, in particular phrenic paralysis or atelectasis were excluded. The evaluation was performed for each hemithorax: 0 = absent, 1 = moderate impairment (diaphragmatic flattening, reduced or paradoxical motion of chest wall and/or diaphragm), 2 = severe impairment (diaphragmatic motion hardly visible).

#### Impairment of respiratory mechanics due to collateral causes

Respiratory motion impairment as collateral finding (prevailing conditions which are not COPD-related, i.e. phrenic paralysis or atelectasis): 0 = absent, 1 = moderate impairment, 2 = severe impairment.

#### Pulmonary trunk ectasia

0 = diameter PA < 29 mm, 1 = diameter PA ≥ 29 mm [18].

#### Right ventricular enlargement

Right ventricle (RV) to left ventricle ratio (LV), 0 = RV/LV ≤ 1.0, 1 = RV/LV > 1.0 [19].

#### Special findings

This category summarizes unspecific findings which are not met by other categories, such as parenchyma bands, dystelectasis, pleural effusion, pleurisy, pneumothorax, or pleural peels. Such special findings were analysed separately for the right and left hemithorax: 0 = absent, 1 = present.

All 39 MRI datasets were evaluated in consensus by three chest radiologists with 2–4 years of experience in pulmonary ^1^H-MRI. MRI2 was assessed one week after MRI1. Every single observation was documented for further evaluation of test-retest reliability. Subscores were calculated for each category as the sum of each lobar score, and the global score was calculated as the sum of all category subscores. Image quality was rated in consensus for every single MRI sequence: 0 = excellent, 1 = reduced but diagnostic, 2 = not diagnostic.

### Statistical analysis

Scores are given as median with interquartile range (Q1 to Q3). The test-retest reliability (MRI1 vs. MRI2) was assessed by weighted kappa with quadratic weighting [20] for ordinal variables and by Cohen´s kappa [21] for nominal variables. We employed the following levels of agreement: 0–0.20 = poor, 0.21–0.40 = fair, 0.41–0.60 = moderate, 0.61–0.80 = substantial, 0.81–1.00 = almost perfect [22]. Global sum scores and category subscores of test and retest were compared by Wilcoxon signed-rank test for skewed distributions and paired Student's t-test for normal distributions. Calculations were done using IBM SPSS statistics 22 (International Business Machines Corporation IBM, Armonk, NY, USA).

## Results

### Technical feasibility and image quality

MRI1 could be completed in 18 of 20 patients, and partially completed in 2 of 20 patients who refused intravenous contrast application. MRI2 was rejected by 1 patient, and again 2 patients refused intravenous application of contrast media. The semiquantitative visual image evaluation did not exceed 20 minutes per dataset. Inspiratory HASTE, free-breathing TrueFISP, as well as dynamic TrueFISP during maximum inspiration and expiration demonstrated excellent image quality (score = 0) at MRI1 and MRI2. Image quality of other MRI sequences was also thoroughly reported as being diagnostic (score = 1) or excellent (score = 0) with few exceptions: post-contrast VIBE showed insufficient image quality due to respiratory motion artifacts in 2 patients at MRI1 (1 in transversal, 1 in coronal plane) and 3 at MRI2 (2 transversal, 1 coronal). The respiratory triggered BLADE showed insufficient image quality in 2 subjects at MRI2 (1 transversal, 1 coronal). The test-retest reliability of image quality scoring of MRI1 vs. MRI2 was moderate with ĸ = 0.41 (95%CI = 0.21–0.60), meaning that image quality may be somewhat variable within the same subject.

### Test-retest reliability of MRI in COPD

The median global score for n = 19 patients was 10 (Q1: 8.00; Q3: 16.00) for MRI1 and 11 (Q1: 6.00; Q3: 15.00) for MRI2. The global scores and category subscores were not significantly different between MRI1 and MRI2 (subscore data not shown). We found an “almost perfect” test-retest reliability of MRI1 vs. MRI2 (ĸ = 0.86, 95%CI = 0.81–0.91) (Table 3) based on the entirety of single observations. Kappa statistics indicated “substantial” or “almost perfect” agreement for MRI1 vs. MRI2 with regard to the majority of category subscores (ĸ = 0.64–1.00) (Table 3). For special findings such as atelectasis or pleural effusion (ĸ = 0.42, 95%CI = 0.00–0.93), the agreement for MRI1 vs. MRI2 was “moderate” (Table 3), for tracheal abnormalities (ĸ = 0.21, 95%CI = 0.00–0.75) it was “fair”.

**Table 3:** Weighted kappa of test vs. retest (consensus of n = 3 readers), displayed for category subscores and for the global score.

Category	κ	95% CI
Tracheal disease	0.21	0.00–0.75
Bronchial disease	0.88	0.55–0.98
Small airway disease	1.00	1.00–1.00
Consolidations	0.80	0.55–1.00
Nodules	0.75	0.32–1.00
Parenchymal defects	0.77	0.49–1.00
Perfusion defects	0.77	0.61–0.93
Perfusion defects (collateral findings)	0.74	0.24–1.00
Impaired respiratory mechanics	0.76	0.45–1.00
Impaired respiratory mechanics (collateral findings)	1.00	1.00–1.00
Pulmonary trunk ectasia	0.86	0.17–1.00
Right ventricular enlargement	0.64	0.00–1.00
Special findings	0.42	0.00–0.93
Global	0.86	0.81–0.91

**Fig 1:**
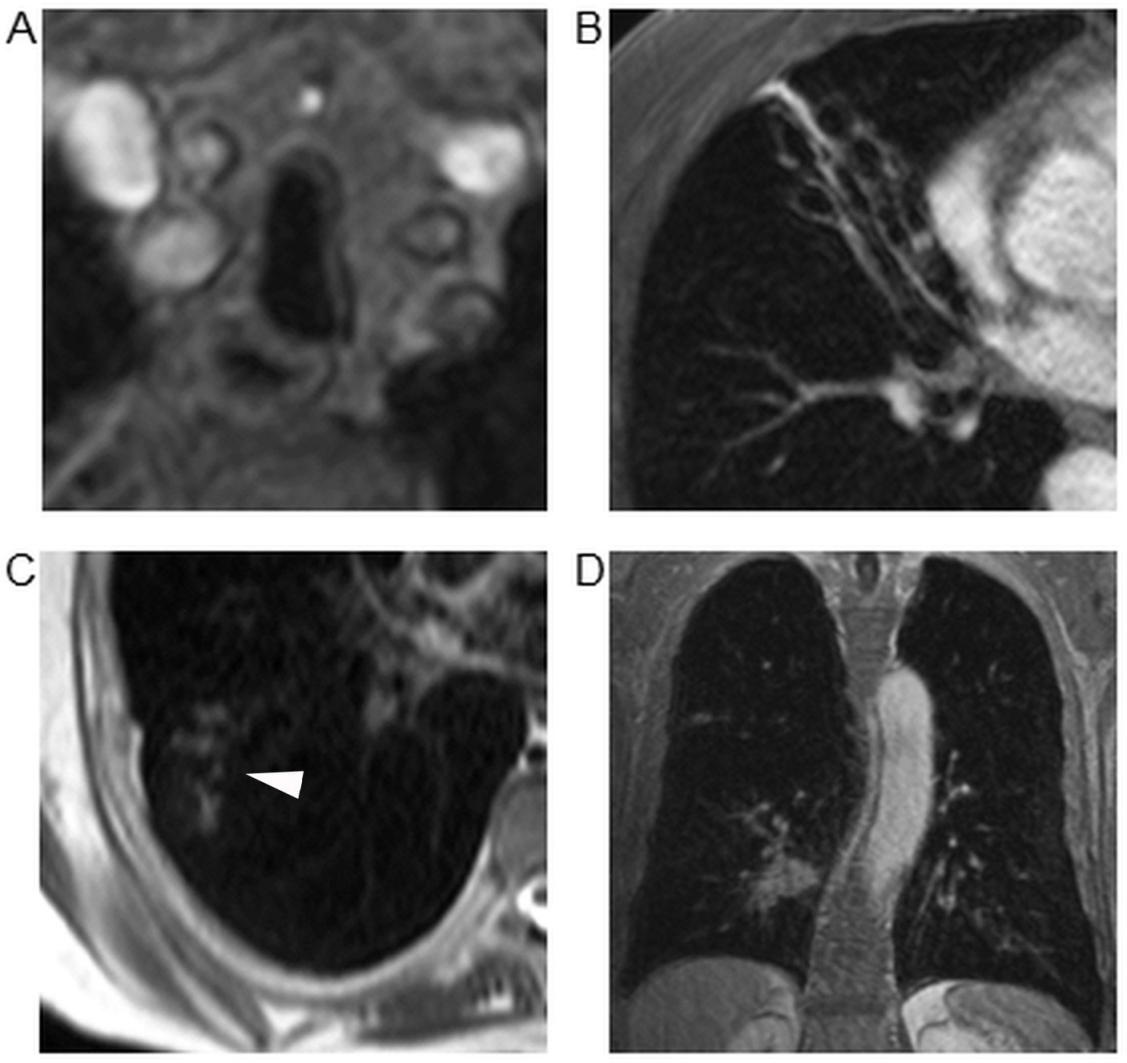
Examples of of typical findings. (A) Saber sheath trachea in a 69 year old patient diagnosed with GOLD stage IV (“tracheal disease”-score = 1). (B) Complete destruction of the middle lobe due to severe bronchiectasis in a 59 year old patient with GOLD stage II (lobar score for “bronchial disease” = 2). (C) Small airway disease (arrowhead) in the right lower lobe of a 50 year old patient with GOLD stage I (lobar score for the category “small airway disease” = 1). (D) Bronchial carcinoma ≥ 1.0 cm in the right lower lobe adjacent to the bronchovascular bundle of a 79 year old patient with GOLD stage IV (lobar score for the category “nodules” = 2).

**Fig 2:**
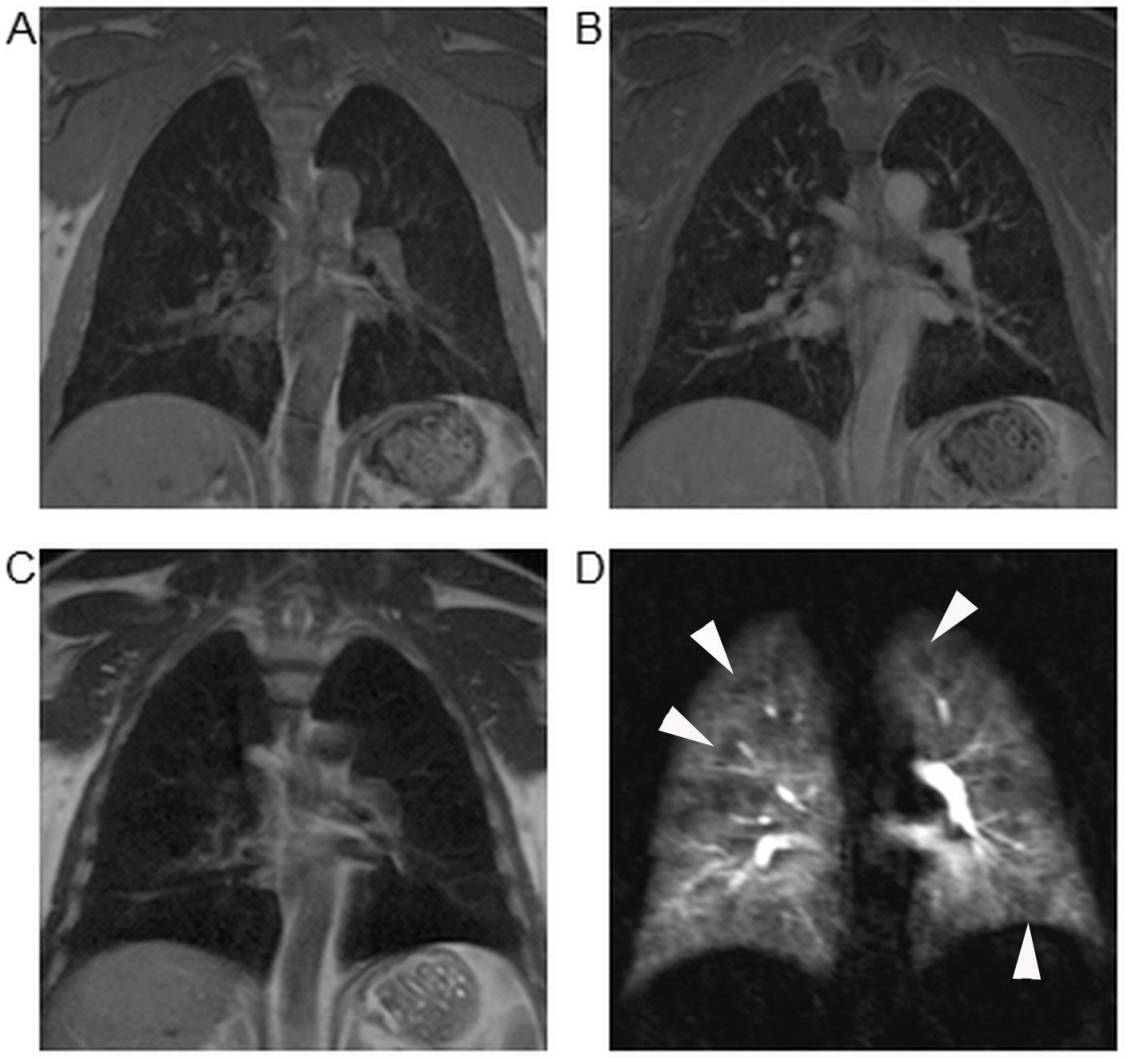
Discrepancy of parenchymal structure and function in mild COPD. 57 year old male patient suffering from mild COPD (GOLD stage I). VIBE (A), post-contrast VIBE (B), as well as HASTE (C) did not show significant parenchymal alterations. However, functional MRI with 4D first pass perfusion revealed scattered minor defects (arrowheads) across the lung on subtracted images (D) and therefore was rated with a perfusion score of 1 for each lobe.

**Fig 3:**
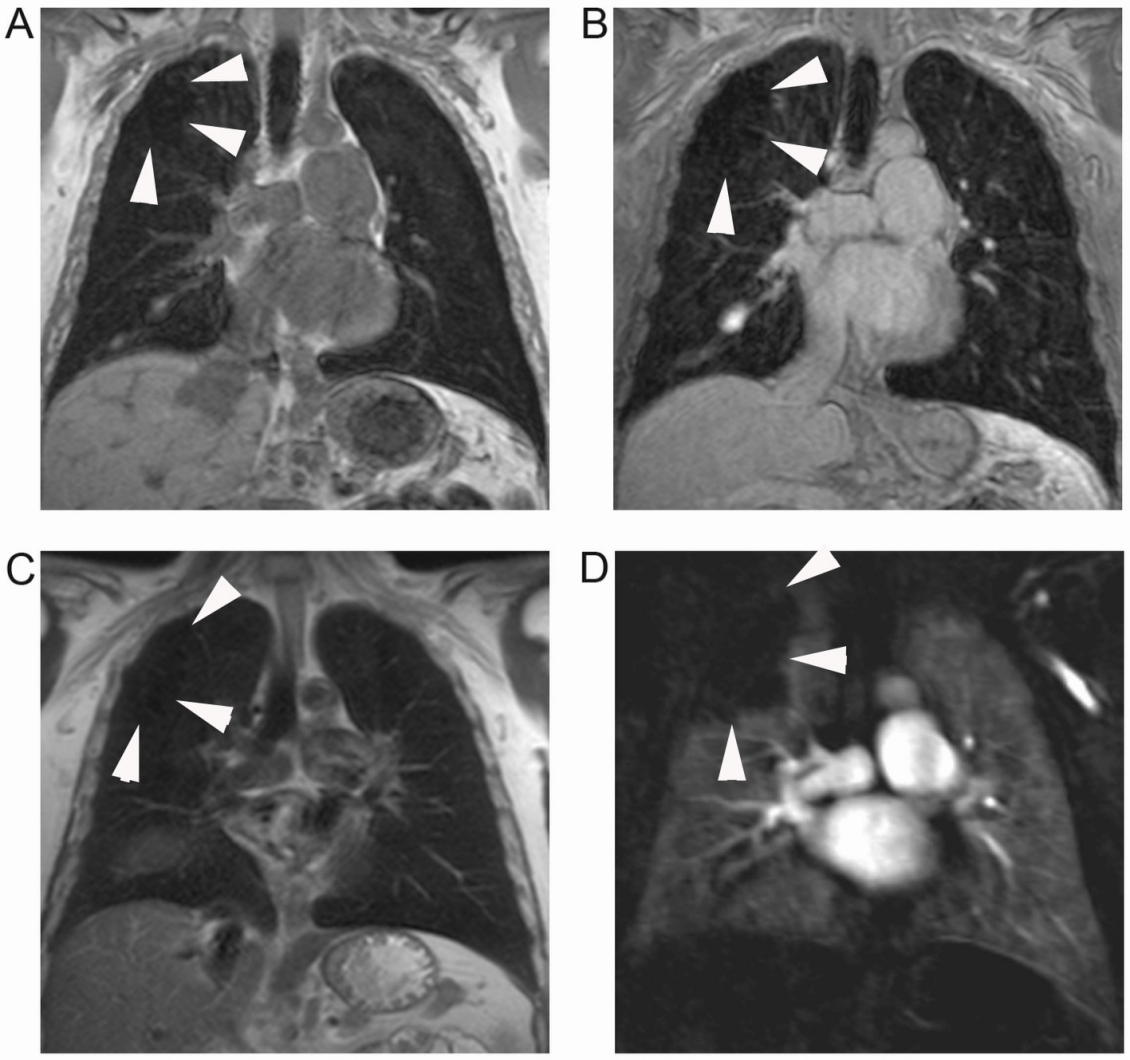
Pulmonary emphysema with concomitant perfusion deficit. Grossly circumscribed structural defect (arrowheads) located in the periphery of the right upper lobe of a 50 year old male patient with GOLD stage I, as displayed on VIBE (A), post-contrast VIBE (B), and HASTE (C). A corresponding, but slightly larger defect (arrowheads) is visible on subtraction images of MR perfusion datasets (D). With regard to the category subscore “parenchymal defects”, the lobe was rated with a score of 1, and with regard to the perfusion score, it was rated with a 2. Additionally, one recognizes a small pleural effusion in the right oblique fissure which is also visible in Fig 4B.

At single patient level, airway abnormalities (Fig 1), pulmonary consolidations, COPD related-perfusion defects (Fig 2, Fig 3), and impairments of respiratory mechanics (collateral findings) were detected in the same number of patients at MRI1 and MRI2 (Table 4). The prevalence of pulmonary nodules was reported slightly higher at MRI1 (37%) than in MRI2 (26%), just as well as the prevalence of special findings (42% vs. 32%). Other categories differed by 1 subject at most between MRI1 and MRI2 (Table 4).

**Table 4:** Number of patients with COPD-relevant MRI findings in MRI1 and MRI2 ordered by categories. 2 individuals rejected contrast media at each examination.

Findings (Category)	MRI1	MRI2
Tracheal disease	3/19	3/19
Bronchial disease	6/19	6/19
Small airway disease	4/19	4/19
Consolidations	6/19	6/19
Nodules	7/19	5/19
Parenchymal defects	9/19	10/19
Perfusion defects	16/17	16/17
Perfusion defects (collateral findings)	3/17	4/17
Impaired respiratory mechanics	8/19	9/19
Impaired respiratory mechanics (collateral findings)	2/19	2/19
Pulmonary trunk ectasia	5/19	4/19
Right ventricular overload	2/19	1/19
Special findings	8/19	6/19

**Fig 4:**
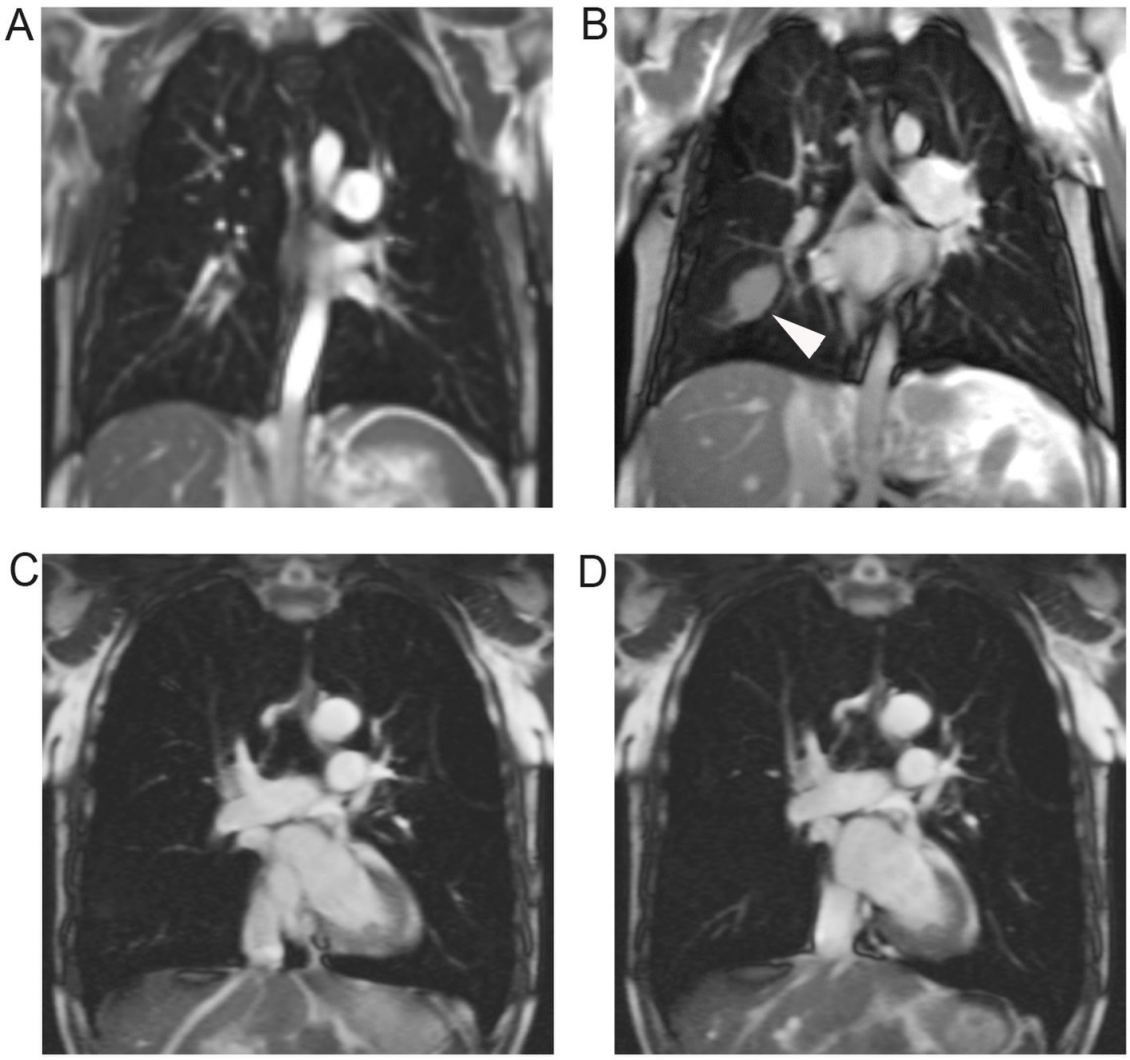
Respiratory mechanics in mild and advanced COPD. Snapshots from dynamic TrueFISP in coronal orientation showing respiratory mechanics in a 50 year old male patient with GOLD stage I (A, B) and a 77 year old male patient with GOLD stage III (C, D). Diaphragmatic function of the patient with mild COPD appeared normal, as shown in inspiration (A) and expiration (B). Note a small pleural effusion in the right oblique fissure as secondary finding (arrowhead). The patient with GOLD stage III showed bilateral phrenic flattening and signs of whipsaw-motion in inspiration (C) and expiration (D). Phrenic motion amplitude was bilaterally restricted, with hardly recognizable motion on the left side (category subscore for respiratory mechanics right = 1; left = 2). Note an emphysemal bulla in the left upper lobe as additional finding.

The number of lung lobes with COPD-related findings was relatively stable for most categories from MRI1 through MRI2, as well as their attributed score value (Table 5). Airway categories, consolidations, nodules, collateral perfusion defects, collateral impairments of respiratory mechanics (Fig 4) and right ventricular enlargement showed similarly low prevalence of up to 16% of lung lobes/chest compartments, mainly with low score values (score = 1) at both timepoints (Table 5). A relatively high number of lung lobes/chest compartments showed parenchymal defects (Fig 3) based on morphological MRI sequences (26% vs. 33%), perfusion defects (83% vs. 84%) and impaired respiratory mechanics (39% vs. 45%) as well as pulmonary trunk ectasia (26% vs. 21%) at both examinations (Table 5). In particular the number of parenchymal defects seemed to differ between both examinations, with a tendency towards a higher detection rate of minor parenchymal defects at MRI2 (37 vs. 29 lobes), whereas the number of large parenchymal defects remained stable.

**Table 5:** Number of abnormal lung lobes or thoracic compartments as observed on MRI1 or MRI2, ordered by category and by score point (0, 1, or 2 points) for n = 19 patients (n = 114 lobes). Note that the total number of lung lobes for the category perfusion is reduced by 2 patients (12 lobes) who rejected contrast media at each examination.

	Number of rated lobes (∑ = 114) / or compartments						
Pathology (Category)	N score = 0		N score = 1		N score = 2		Compartment
	MRI1	MRI2	MRI1	MRI2	MRI1	MRI2	
Tracheal disease	16	16	3	3	0	0	Trachea / Stem
Bronchial disease	106	107	7	5	1	2	Lobe
Small airway disease	110	110	4	4	0	0	Lobe
Consolidations	108	107	5	5	1	2	Lobe
Nodules	104	106	8	6	2	2	Lobe
Parenchymal defects	84	76	29	37	1	1	Lobe
Perfusion defects (102 lobes)	17	16	49	46	36	40	Lobe
Perfusion defects (collateral findings, 102 lobes)	99	98	2	2	1	2	Lobe
Impaired respiratory mechanics	23	21	13	14	2	3	Chest r/l
Impaired respiratory mechanics (collateral findings)	36	36	2	2	0	0	Chest r/l
Pulmonary trunk ectasia	14	15	5	4			Pulm. trunk
Right ventricular enlargement	17	18	2	1			Heart
Special findings	29	32	9	6			Chest r/l

## Discussion

The aim of the study was the assessment the test-retest reliability of a combined morpho-functional MRI protocol based on commercially available sequences for COPD-related regional abnormalities of lung structure, perfusion, and respiratory mechanics as well as pulmonary hypertension. Continuously high image quality is a prerequisite for this concept, since insufficient or varying image quality decreases reproducibility with significant influence on image-based quantitative or semiquantitative biomarkers. Many patients with severe COPD are short of breath and thus unable to comply with long breath-holding times. Image quality is therefore frequently limited. Gay et al. reported a maximum breath-holding time of 25 seconds during CT imaging of patients with COPD, chronic heart failure or extensive smoking history and 45 seconds in patients without pulmonary disease [23]. To meet the requirements for scanning COPD patients, the protocol employed respiratory triggered and free breathing sequences which are less stressful for the patient and reduce or eliminate the influence of respiratory motion. Breath-holding times of most sequences were below 25 seconds with few exceptions: The 4D perfusion sequence (TWIST) covered a time period of 37 seconds since circulation times are difficult to predict in morbid patients and frequently prolonged. However, observed individual abilities of COPD patients to hold breath were sufficient for artifact-free perfusion acquisition during the first pulmonary passage of the contrast bolus (15–25s). The fat-saturated VIBEs were the only morphological acquisitions exceeding breath-holding times of 25 seconds. In general, the imaging protocol proved to be robust since image quality was at least diagnostic at both examinations, with only few fat-saturated VIBEs and BLADE acquisitions in different patients showing insufficient image quality. This may be partially due to the prolonged breath-holding time of fat-saturated VIBE, but advances in parallel imaging will certainly allow for a reduction of fat-saturated VIBE acquisition times in the near future. Although most MRI data sets showed a high level of image quality, the test-retest reliability of image quality scoring of MRI1 vs. MRI2 was only moderate with ĸ = 0.41. This reflects intra-subject fluctuations of image quality related to image artifacts of insignificant extent, mainly minor respiratory motion, cardiac motion, and in some cases, Gibbs ringing artifacts. Furthermore, the 4D DCE perfusion sequence and the contrast regimen were also designed to meet the requirements of a software-based calculation of regional quantitative perfusion parameters. The designed MRI protocol also met the expectations concerning test-retest reliability, since COPD-related lung abnormalities were depicted with “almost perfect” reproducibility within 1 day. For imaging-based phenotyping of COPD, the extent of airway remodeling and pulmonary emphysema would need to be systematically assessed [24]. Today, CT is the modality of choice for in vivo imaging of the pulmonary parenchyma and airways in COPD [2,25]. MRI of the lung remains challenging due to low proton density and thus low MRI signal of lung tissue. Spatial resolution of MRI is inferior to CT in the order of 2 to 4 times, thus affecting airway imaging which can be performed down to the segmental level in healthy individuals. However, bronchial wall thickening and dilatation increase visibility on MRI and improve the specificity of MRI for airway pathology. Furthermore, small airways disease with tree-in-bud pattern is also sensitively depicted by MRI [6,26]. The present study is the first of its kind to systematically evaluate airway alterations in COPD patients with MRI. Airway pathologies were found in approximately half of the patients in both MR examinations. However, the number of affected lung lobes was infrequent at both time points. Kappa statistics indicated “almost perfect” reproducibility for abnormalities of large and small airways. The evaluation of tracheal abnormalities showed a “fair” agreement between MRI1 and MRI2, reflecting reduced test-retest reliability compared to other subscore categories. This might be based on the fact that the evaluation of tracheal narrowing was based on the combined evaluation of free-breathing, respiratory-triggered and inspiratory breath-hold acquisitions which comes close to but obviously cannot replace dynamic acquisitions during forced inspiration/expiration and free breathing. In patients with COPD, the additional loss of signal due to emphysematous destruction, hyperinflation and hypoxic pulmonary vasoconstriction deteriorates conditions for structural ^1^H-MRI [27,28]. In the present study, parenchymal defects based on morphological MR images were detected in a substantial number of lung lobes at both MRI1 and MRI2 (26% vs. 33%) with “substantial” agreement between both examinations. Ley-Zaporozhan et al. compared MRI-based and CT-based emphysema classification and reported that the extent of emphysema was rated correctly using morphological MRI sequences in 63% of lung lobes, suggesting that emphysema characterization with structural MRI is less sensitive than with CT, but provides a certain impression of emphysema severity [14]. Moreover, the protocol was aimed to cover frequent COPD-related inflammatory or potentially malignant comorbidities such as pneumonia and pulmonary nodules, which should not be missed in cross-sectional imaging of the lung. These findings had a low prevalence in our population but were depicted with high (“substantial”) test-retest reproducibility. This is perfectly in keep with recently published results on lung nodule detection in a lung cancer CT screening population by Sommer et al., who could show that the inter-rater variability for lung nodules was only 84% with κ = 0.65 with a similar morphological protocol but without contrast application [29]. In COPD patients, perfusion can be adversely affected by hypoxic pulmonary vasoconstriction, remodeling or rarefication of the pulmonary vasculature due to tissue destruction [30]. MRI is capable of depicting such perfusion abnormalities with similar sensitivity and specificity as pulmonary radionuclide scintigraphy [31]. Moreover, Ley-Zaporozhan et al. reported a high sensitivity of MR perfusion imaging for emphysematous lung areas visible on CT [32]. However, contrast enhanced MR perfusion has been shown to be an appropriate surrogate parameter for both, parenchymal destruction and airway obstruction [33–35]. The 4D DCE perfusion study employed for the present study demonstrated “substantial” test-retest reproducibility concerning the presence, localization and extent of lung areas with reduced perfused blood volume. Additionally, it revealed a high prevalence of lung areas with reduced blood content per volume at both timepoints (86% vs. 88%). It is well known that advanced hyperinflation and emphysema in COPD affect chest wall and diaphragmatic motion [16,17] leading to reduced extent of motion and/or paradoxical movement, which has been studied by 4DCT [36] as well as time-resolved MRI acquisitions [15,17]. Image-based evaluation of respiratory mechanics is of potential value for patient monitoring in the context of interventions such as lung volume reduction surgery, which are believed to improve lung function through respiratory mechanics [37]. However, the reproducibility of findings in dynamic TrueFISP during maximum inspiration and expiration has not been assessed yet. Dynamic TrueFISP sequences during maximum inspiration and expiration revealed respiratory motion impairment in the same number of chest compartments at MRI1 and MRI2 (each 17/38) with similar extent of severity. In this context, extensive motivation and guidance of the patients ensured high test-retest reliability. Morphological indicators of pulmonary hypertension were detected with high reproducibility, in detail right ventricular enlargement and pulmonary trunk ectasia, thereby providing information on the probability of severe exacerbations [38] and 5-year survival rate [39]. Pathologies addressed by the category “special findings” (which entailed mainly small atelectasis and minor pleural effusion) are per se highly variable throughout 24 hours. As a result, kappa statistics indicated moderate test-retest reliability for this category, which should not be attributed to MRI. In the near future, new MRI techniques based on near zero echo times such as ultra-short echo time (UTE) sequences will improve image quality of proton MRI of the lung, providing higher spatial resolution, better signal-to-noise ratio and shorter scan time [40, 41]. Most clinical scanner vendors already offer UTE sequences for selected MRI devices, in many cases as work in progress (WIP) sequences. First experiences in subjects with cystic fibrosis [42] indicate the high potential of these techniques in the evaluation of pulmonary structure and function in subjects with lung diseases. The proposed imaging protocol will be the basis of a prospective study (DRKS00005072) comparing the performance of MRI- with CT-based phenotyping of COPD in a large multicenter subcohort of the German COPD cohort (Impact of Systemic Manifestations/Comorbidities on Clinical State, Prognosis, Utilisation of Health Care Resources in Patients with COPD study “COSYCONET”, NCT01245933).

## Conclusion

The proposed imaging protocol allowed for the evaluation of COPD-related alterations of bronchi, pulmonary parenchyma, perfusion, and respiratory mechanics with high test-retest reliability. Even in patients with advanced COPD, image quality of most acquisitions was at least diagnostic at both examinations. The proposed imaging protocol appears valuable for phenotyping COPD as well as monitoring of regional lung structure and function in clinical trials.
